# A Novel Analytical Design Technique for a Wideband Wilkinson Power Divider Using Dual-Band Topology

**DOI:** 10.3390/s21196330

**Published:** 2021-09-22

**Authors:** Asif I. Omi, Rakibul Islam, Mohammad A. Maktoomi, Christine Zakzewski, Praveen Sekhar

**Affiliations:** 1 Department of Engineering & Computer Science, Washington State University Vancouver, Vancouver, WA 98686, USA; asif.omi@wsu.edu; 2 Department of Electrical & Computer Engineering, University of Illinois at Urbana-Champaign, Urbana, IL 61801, USA; rakibul2@illinois.edu; 3 Department of Physics & Engineering, The University of Scranton, Scranton, PA 18510, USA; mohammad.maktoomi@scranton.edu (M.A.M.); christine.zakzewski@scranton.edu (C.Z.)

**Keywords:** coupled-line, coupler, dual-band, S-parameter, Wilkinson power divider

## Abstract

In this paper, a novel analytical design technique is presented to implement a coupled-line wideband Wilkinson power divider (WPD). The configuration of the WPD is comprised of three distinct coupled-line and three isolation resistors. A comprehensive theoretical analysis is conducted to arrive at a set of completely new and rigorous design equations utilizing the dual-band behavior of commensurate transmission lines. Further, the corresponding S-parameters equations are also derived, which determine the wideband capability of the proposed WPD. To validate the proposed design concept, a prototype working at the resonance frequencies of 0.9 GHz and 1.8 GHz is designed and fabricated using 60 mils thick Rogers’ RO4003C substrate. The measured result of the fabricated prototype exhibits an excellent input return loss > 16.4 dB, output return loss > 15 dB, insertion loss < 3.30 dB and a remarkable isolation > 22 dB within the band and with a 15 dB and 10 dB references provide a fractional bandwidth of 110% and 141%, respectively.

## 1. Introduction

Power divider/splitter circuits are one of the essential building blocks in all modern microwave and wireless communication systems [1]. The three-port Wilkinson power divider (WPD) is considered a paragon among such passive components. WPD was first published in 1960 by Ernest J Wilkinson and used to provide electrically isolated output branches with the same phase and equal power division, with all terminated ports matched, in a very simple layout [2]. However, the presence of the intrinsically narrow bandwidth of the conventional WPD has turned out to be one of the major design challenges. A variety of modified WPD configurations have emerged over the years to cater to the needs of wideband, harmonic-suppressed, and multi-standard topologies [3,4,5,6,7,8,9,10,11,12,13,14,15,16,17,18,19,20,21,22,23,24,25,26,27]. Recently, the wideband WPDs have gained paramount importance in various applications such as power amplifiers [28,29,30,31], Hilbert transformer-based dual-band single sideband modulator [32,33], antenna arrays [34], phase shifters [35], and other RF front-end systems [36]. This is mainly due to the recent progress in high data-rate 5G/6G wireless systems that has refueled significant research interest in developing the next generation of wideband power dividers [37,38,39,40,41,42,43,44,45,46,47,48].

**Figure 1:**
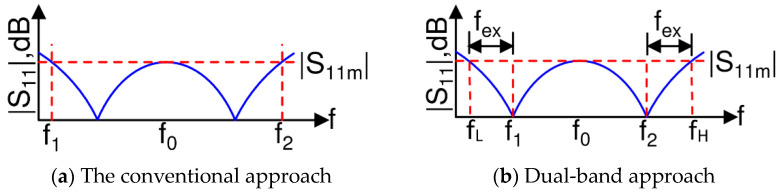
The conventional vs. the proposed design methodologies.

To broaden the operational bandwidth, the fundamental idea has been to incorporate a multi-section topology as proposed by Cohn [3]. Many different varieties of broadband power dividers ensued [4,5,6,7,8,9,10,11,12] following Cohn’s idea. The conventional multi-section WPD design [2] is a fully analytical technique but only for a two-section WPD. For three and more sections, the reported technique is a table-based design and is, therefore, less computer friendly. Furthermore, among the multiple other demonstrated techniques to extend the bandwidth, the notable ones are those based on the stepped-impedance matching network [13], complementary conducting-strip transmission line (CCSTL) [14], port extension [15,16,17], substrate-integrated waveguide (SIW) [18], series/parallel RLC networks [19,20,21,22], composite right-/left-handed transmission (CRLT) lines [23], and optimized isolation network [24,25,26,27]. While CCSTL and SIW suggest alternate structure to enhance the bandwidth, on the contrary the lumped RLC networks, CRLT lines as well as the isolation network add complexity in theoretical analysis with an increase in length to serve the same purpose. Most of these works only focus on improving the bandwidth performance for either the input/output reflection parameters or the insertion loss parameter or the isolation parameter or their combinations, but not all together. In addition, for all these conventional designs for a BW = *f*_2_ − *f*_1_ the reflection profile appears like the one shown in Figure 1a with *f*_0_ being the mid-band frequency and −|*S*_11*m*_| being the minimum return loss. Therefore, in the event of ubiquitous component/process variations, the bandwidth target is never achieved, necessitating post-design optimization.

In this paper, a new and rigorous analytical design methodology for the classic tri-section cascaded coupled-line-based topology is presented to maximize the bandwidth performance for all the S-parameters simultaneously. Furthermore, as depicted in Figure 1b, the proposed design methodology utilizes a dual-band design concept [37] to arrive at a wideband design. This dual-band design approach guarantees that the resonance frequencies are always located at *f*_1_ and *f*_2_, and therefore, the achieved bandwidth is BW = *f_H_* − *f_L_* = (*f*_2_ − *f*_1_) + 2*f_ex_*, where 2*f_ex_* is the extra bandwidth that provides a margin for process/component variations. This approach is the usual choice for a commercial development setup, where the minimum bandwidth requirement *f*_2_ − *f*_1_ will always be met, and 2*f_ex_* will allow flexibility to meet design goal requirements.

The remainder of this paper is organized as follows. In Section 2, the theory and design equations of the proposed WPD are developed. The derivation of the corresponding S-parameters and bandwidth performance is presented in Section 3. Then, the complete circuit of the proposed WPD along with its step-by-step design procedure is explained in Section 4 and few examples are demonstrated to support the theoretical design capability. Afterward, the fabricated prototype and its EM simulated and measured results are demonstrated in Section 5. Finally, the performance of this work is compared with some of the state-of-the-art techniques followed by a conclusion in Section 6.

## 2. The Proposed Analytical Design Equations of the WPD

**Figure 2:**
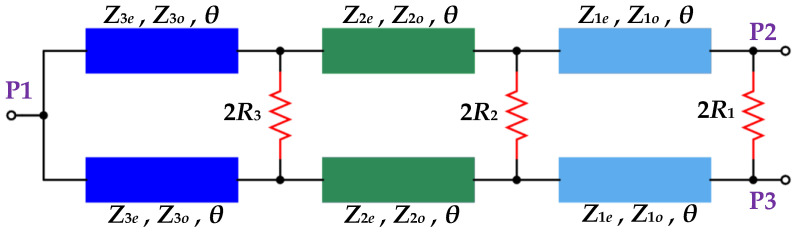
The tri-section coupled-line WPD.

A tri-section coupled-line 3-dB Wilkinson power divider (WPD) configuration is depicted in Figure 2. P1 denotes the input port of the WPD whereas P2 and P3 denote the two output ports. Each of the ports is terminated with a real impedance *Z*_0_ (normally equal to 50 Ω). Further, *Z_ke_* and *Z_ko_* refer to the even and odd mode impedances of the *k*th section coupled-line while *θ* refers to their electrical length with *k* ϵ {1, 2, 3}. As all the transmission lines are coupled, the physical space between the two arms of the divider will normally be very close, resulting in a miniaturized structure. 

The coupling coefficient, *C_k_* of the *k*th section coupled-line is defined as [1]:

(1) $$C_{k}=20\log\frac{Z_{ke}-Z_{ko}}{Z_{ke}+Z_{ko}}$$

As explained in [37], *θ* at *f*_1_ for a dual-band design is chosen as:

(2) $$\theta =\frac{\pi }{1+r}$$

where *f*_1_ and *f*_2_ refer to the minimum band edge frequencies for the bandwidth requirement and *r*= *f*_2_*/f*_1_ is the band ratio.

**Figure 3:**
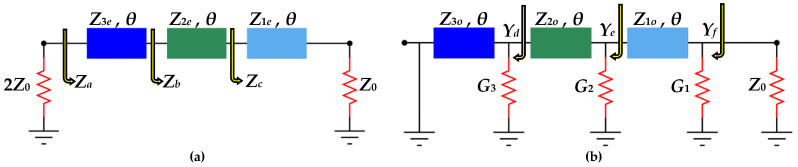
(**a**) The even-mode equivalent circuit, (**b**). The odd-mode equivalent circuit.

Moreover, the three resistors 2*R*_3_, 2*R*_2_, and 2*R*_1_ shown in Figure 2 ensure isolation between port 2 and port 3. Since the WPD shown in Figure 2 is symmetric about a horizontal axis passing through P1, the even/odd mode analysis technique can be utilized for its analysis. The resulting equivalent circuits are depicted in Figure 3.

### 2.1. Even-Mode Analysis

The even-mode equivalent circuit is shown in Figure 3a. This circuit has been obtained from Figure 2 by assuming an open circuit along the axis of symmetry. As shown in the figure, the parameters *Z_a_*, *Z_b_*, and *Z_c_* are the input impedances looking towards the right beginning at Section 3. Using the formula of the input impedance of a transmitted transmission line [1], the following expressions can be written:

(3) $$Z_{c}=Z_{1e}\frac{Z_{0}+jaZ_{1e}}{Z_{1e}+jaZ_{0}}$$

(4) $$Z_{b}=Z_{2e}\frac{Z_{c}+jaZ_{2e}}{Z_{2e}+jaZ_{c}}$$

(5) $$Z_{a}=Z_{3e}\frac{Z_{b}+jaZ_{3e}}{Z_{3e}+jaZ_{b}}$$

where,

*a* = tan*θ* (6)

Now, substituting the expressions of *Z_c_* into that of *Z_b_* and subsequently the expression of *Z_b_* into that of *Z_a_*, and finally invoking *Z_a_* = 2*Z*_0_ for ideal input matching condition, the following equations are obtained [37].

(7) $$Z_{2e}^{2}+\frac{Z_{2e}}{aZ_{3e}}\left[aZ_{3e}^{2}+Z_{3e}X_{1e}-4aZ_{0}^{2}(1-b)\right]-\left[4Z_{0}^{2}(1-b)+aZ_{3e}X_{1e}\right]=0$$

(8) $$a^{2}\left(1-b\right)Z_{3e}^{2}+Z_{3e}\left(bZ_{2e}-aX_{1e}\right)-aZ_{2e}\left(X_{1e}+aZ_{2e}\right)=0$$

where,

(9) $$b=1-\frac{R_{1e}}{2Z_{0}}$$

(10) R1e=Re{Zc}=Z0Z1e2(1+a2)Z1e2+Z02a2

(11) X1e=Im{Zc}=aZ1e(Z1e2−Z02)Z1e2+Z02a2

Now eliminating *Z*_2*e*_ from (7) and (8) results in a 4th-order equation in *Z*_3*e*_:

(12) $$AZ_{3e}^{4}+BZ_{3e}^{3}+CZ_{3e}^{2}+DZ_{3e}+E=0$$

where,

(13) $$A=a^{2}b(1-b)$$

(14) $$B=2a^{3}(1-b)X_{1e}$$

(15) $$C=2b^{2}R_{1e}Z_{0}-X_{1e}^{2}(a^{4}+2a^{2}+b)$$

(16) $$D=-4Z_{0}R_{1e}X_{1e}a^{3}$$

(17) $$E=2Z_{0}R_{1e}a^{2}(X_{1e}^{2}-2bR_{1e}Z_{0})$$

Subsequently, *Z*_2*e*_ is obtained from (7) and (8) as follows.

(18) $$Z_{2e}=\frac{aZ_{3e}[a(1-b)(4Z_{0}^{2}-Z_{3e}^{2})+(1+a^{2})X_{1e}Z_{3e}]}{(b+a^{2})Z_{3e}^{2}-4Z_{0}^{2}(1-b)a^{2}}$$

The even-mode design is completed by choosing a suitable value of *Z*_1*e*_—thus those of *R*_1*e*_ and *X*_1*e*_—and then finding *Z*_3*e*_ from (12) and *Z*_2*e*_ from (18). Out of the four roots of *Z*_3*e*_ obtained from (12) using MATLAB, only a positive real value for *Z*_3e_ and *Z*_2e_ lying between 20 Ω to 120 Ω is considered for modeling and implementation in the microstrip technology. The criteria for choosing *Z*_1*e*_ will be outlined later in Section 3 and Section 4.

### 2.2. Odd-Mode Analysis

The odd-mode equivalent circuit is shown in Figure 3b. This circuit has been obtained from the one shown in Figure 2 by assuming a short-circuit along the axis of symmetry. Due to the presence of shunt resistors, it is easier to work in terms of admittances, therefore, *G_i_* and *Y_io_* are defined as *G_i_* = 1/*R_i_* and *Y_io_* = 1/*Z_io_*, *i* ϵ {1, 2, 3}.

As shown in Figure 3b, the parameters *Y_f_*, *Y_e_*, *Y_d_* are the input admittances looking towards the left at the *i*th section including the conductance *G_i_* = 1/*R_i_* at that node. Applying the formula of input admittance for a terminated transmission line section [1], we have:

(19) $$Y_{d}=G_{3}-j\frac{Y_{3o}}{a}$$

(20) $$Y_{e}=G_{2}+Y_{2o}\frac{Y_{d}+jaY_{2o}}{Y_{2o}+jaY_{d}}$$

(21) $$Y_{f}=G_{1}+Y_{1o}\frac{Y_{e}+jaY_{1o}}{Y_{1o}+jaY_{e}}$$

Now, substituting the expression of *Y_d_* into that of *Y_e_* and subsequently the expression of *Y_e_* into that of *Y_f_*, and finally invoking *Y_f_* = 1/*Z*_0_ = *Y*_0_ for the ideal output matching condition, the following equations are obtained.

(22) $$P_{1}G_{2}+Q_{1}G_{3}+S_{1}G_{2}G_{3}+T_{1}=0$$

(23) $$P_{2}G_{2}+Q_{2}G_{3}+S_{2}G_{2}G_{3}+T_{2}=0$$

where,

(24) $$P_{1}=a\left(Y_{2o}+Y_{3o}\right)\left(G_{1}-Y_{o}\right)$$

(25) $$Q_{1}=a\left(Y_{1o}+Y_{2o}\right)\left(G_{1}-Y_{o}\right)$$

(26) $$S_{1}=a\left(Y_{1o}\right)$$

(27) $$T_{1}=aY_{1o}Y_{2o}\left(Y_{1o}+Y_{2o}\right)+Y_{1o}Y_{3o}\left(aY_{1o}-\frac{Y_{2}}{a}\right)$$

(28) $$P_{2}=Y_{1o}\left(Y_{2o}+Y_{3o}\right)$$

(29) $$Q_{2}=Y_{1o}Y_{2o}-a^{2}Y_{1o}{}^{2}$$

(30) $$S_{2}=a^{2}\left(Y_{o}-G_{1}\right)$$

(31) $$T_{2}=\left(G_{1}-Y_{o}\right)\left(Y_{1o}Y_{2o}+Y_{2o}Y_{3o}+Y_{1o}Y_{3o}-a^{2}Y_{2o}{}^{2}\right)$$

Now eliminating *G*_3_ from (22) and (23) results in a second-order equation in *G*_2_:

(32) $$MG_{2}^{2}+NG_{2}+L=0$$

where,

(33) $$M=P_{2}S_{1}-P_{1}S_{2}$$

(34) $$N=P_{2}Q_{1}-P_{1}Q_{2}+T_{2}S_{1}-T_{1}S_{2}$$

(35) $$L=T_{2}Q_{1}-T_{1}Q_{2}$$

From (32), *G*_2_ can be found as follows:

(36) $$G_{2}=\frac{-N\pm \sqrt{N^{2}-4ML}}{2M}$$

Putting the values of (36) in (22), *G*_3_ can be found as follows:

(37) $$G_{3}=\frac{-\left(T_{1}+G_{2}P_{1}\right)}{Q_{1}+G_{2}S_{1}}$$

Choosing suitable values of *G*_1_, *Y*_1*o*_, *Y*_2*o*_, and *Y*_3*o*_, and subsequently finding the values *G*_2_ from (36) and that of *G*_3_ from (37) completes the WPD design process. The criteria for choosing the free variables *G*_1_, *Y*_1*o*_, *Y*_2*o*_, *Y*_3*o*_ will be outlined later in Section 3 and Section 4.

## 3. The Scattering Parameters of the WPD and BW Determination

The expression developed in Section 2 can be used to analytically design the three-section coupled-line WPD. However, due to the presence of many free variables, it is pertinent to find the expression of the S-parameters of the WPD. Due to the symmetry of the circuit shown in Figure 1, the expressions of the S-parameters for the WPD are related to the S-parameters of equivalent even-odd mode circuits as follows [16].

(38) $$S_{11}=S_{11e}$$

(39) $$S_{12}=S_{21}=S_{31}=S_{13}=\frac{1}{\sqrt{2}}S_{21e}$$

(40) $$S_{22}=S_{33}=\frac{1}{2}(S_{22e}+S_{22o})$$

(41) $$S_{23}=S_{32}=\frac{1}{2}(S_{22e}-S_{22o})$$

From (38) and (39), it is observed that the input matching and transmission performance of the proposed power divider in Figure 1 is only determined by the even-mode parameters *S*_11*e*_ and *S*_21*e*_. However, the output matching and isolation performances are affected by both the even as well as the odd-mode parameters *S*_22*e*_ and *S*_22*o*_ as seen from (40) and (41).

### 3.1. Expressions of Even-Mode S-Parameters

From the even-mode circuit shown in Figure 3a, *S*_11*e*_, *S*_21*e,*_ and *S*_22*e*_ are obtained as follows [1]:

(42) $$S_{11e}=\frac{Z_{a}-2Z_{0}}{Z_{a}+2Z_{0}}=\frac{X+Y}{A_{e}+\frac{B_{e}}{Z_{0}}+2C_{e}Z_{0}+2D_{e}}$$

(43) $$S_{21e}=\frac{2\sqrt{2}S_{11e}}{W\left(X+Y\right)}$$

(44) $$S_{22e}=\frac{S_{11e}\left(X-Y\right)}{\left(X+Y\right)}$$

where, *Z_a_* can be calculated utilizing (3), (4), and (5) after finding *Z*_1*e*_, *Z*_2*e*_, and *Z*_3*e*_. Further, the parameters *W*, *X*, *Y*, *A_e_*, *B_e_*, *C_e_*, and *D_e_* can be calculated from the conversion between ABCD-parameters and S-parameters as follows [1]:

(45) $$X=\frac{B_{e}}{Z_{0}}-2C_{e}Z_{0}$$

(46) $$Y=A_{e}-2D_{e}$$

(47) W=(1+a2)−32

(48) $$A_{e}=\left[1-a^{2}(u+v+\frac{v}{u})\right]$$

(49) $$B_{e}=jaZ_{1e}\left[1+u+v-a^{2}(\frac{v}{u})\right]$$

(50) $$C_{e}=ja\frac{1}{Z_{1e}}\left[1+\frac{1}{u}+\frac{1}{v}-a^{2}(\frac{u}{v})\right]$$

(51) $$D_{e}=\left[1-a^{2}(\frac{1}{u}+\frac{1}{v}+\frac{u}{v})\right]$$

(52) $$u=\frac{Z_{2e}}{Z_{1e}}$$

(53) $$v=\frac{Z_{3e}}{Z_{1e}}$$

### 3.2. Expression of Odd-Mode S-Parameters

From the odd-mode circuit in Figure 3b, *S*_22*o*_ is obtained as follows:

(54) $$S_{22o}=\frac{\left(1/Y_{f}\right)-Z_{0}}{\left(1/Y_{f}\right)+Z_{0}}$$

where *Y_f_* can be obtained from (21) after finding *Z*_1*o*_, *Z*_2*o*_, *Z*_3*o*_, *G*_1_, *G*_2_, and *G*_3_.

### 3.3. Maximum Input Return loss (RL) between f_1_ and f_2_

To achieve the maximum possible BW between *f_L_* and *f_H_* (as shown in Figure 1), it is desirable to minimize the value of return loss between *f*_1_ and *f*_2_. To that end, using (38), (42), (45), (46), and (48)–(51) we obtain the following expression for *S*_11_:

(55) $$S_{11}=\frac{Z_{0}(k_{1}a^{2}-1)+j(k_{2}a-k_{3}a^{3})}{Z_{0}(3-k_{4}a^{2})+j(k_{5}a-k_{6}a^{3})}$$

where,

(56) $$k_{1}=2(\frac{1}{u}+\frac{1}{v}+\frac{u}{v})-(u+v+\frac{v}{u})$$

(57) $$k_{2}=Z_{1e}(1+u+v)-\frac{2Z_{0}{}^{2}}{Z_{1e}}(1+\frac{1}{u}+\frac{1}{v})$$

(58) $$k_{3}=Z_{1e}(\frac{v}{u})-\frac{2Z_{0}{}^{2}}{Z_{1e}}(\frac{u}{v})$$

(59) $$k_{4}=2(\frac{1}{u}+\frac{1}{v}+\frac{u}{v})+(u+v+\frac{v}{u})$$

(60) $$k_{5}=Z_{1e}(1+u+v)+\frac{2Z_{0}{}^{2}}{Z_{1e}}(1+\frac{1}{u}+\frac{1}{v})$$

(61) $$k_{6}=Z_{1e}(\frac{v}{u})+\frac{2Z_{0}{}^{2}}{Z_{1e}}(\frac{u}{v})$$

Now expressing |*S*_11_| in dB, (55) can be written as

(62) $${\left|S_{11}\right|}_{dB}=10\log\frac{Z_{0}{}^{2}{\left(k_{1}a^{2}-1\right)}^{2}+a^{2}{\left(k_{2}-k_{3}a^{2}\right)}^{2}}{Z_{0}{}^{2}{\left(3-k_{4}a^{2}\right)}^{2}+a^{2}{\left(k_{5}-k_{6}a^{2}\right)}^{2}}$$

To find the maxima/minima of |*S*_11_|dB between *f*_1_ and *f*_2_,

(63) $$\begin{array}{l} \frac{d}{da}{\left|S_{11}\right|}_{dB}=0\\ \Rightarrow c_{1}{\left(a^{2}\right)}^{4}+c_{2}{\left(a^{2}\right)}^{3}+c_{3}{\left(a^{2}\right)}^{2}+c_{4}a^{2}+c_{5}=0 \end{array}$$

where,

(64) $$c_{1}=Z_{0}^{2}(k_{3}^{2}k_{4}^{2}-k_{1}^{2}k_{6}^{2})-2k_{3}^{2}k_{5}^{2}+2k_{3}k_{6}(2k_{2}k_{6}-k_{3}k_{5})$$

(65) $$c_{2}=4Z_{0}^{2}(k_{6}^{2}k_{1}-3k_{3}^{2}k_{4})+2(k_{3}^{2}k_{5}^{2}-k_{2}^{2}k_{6}^{2})$$

(66) $$\begin{array}{l} c_{3}=2Z_{0}^{4}k_{1}k_{4}(k_{4}-3k_{1})+Z_{0}^{2}(k_{1}^{2}k_{5}^{2}-k_{2}^{2}k_{4}^{2}+27k_{3}^{2}+\\ 12k_{2}k_{3}k_{4}-4k_{1}k_{5}k_{6}-3k_{6}^{2})+2k_{5}(k_{2}^{2}k_{6}-k_{2}k_{3}k_{5}) \end{array}$$

(67) $$c_{4}=2Z_{0}^{4}(9k_{1}^{2}-k_{4}^{2})+4Z_{0}^{2}(k_{5}k_{6}-9k_{2}k_{3})$$

(68) $$c_{5}=6Z_{0}^{4}(k_{4}-3k_{1})+Z_{0}^{2}(9k_{2}^{2}-k_{5}^{2})$$

Evaluating *c*_1_, *c*_2_, *c*_3_, *c*_4_, and *c*_5_ from (64) to (68) and subsequently solving (63), we get the values of parameter *a*, which, upon substitution into (62), gives the maxima/minima of |*S*_11_|_*dB*_ between *f*_1_ and *f*_2_. It must be noted that earlier we defined the expression for *a* in (6), which is a particular solution of (63) and indicates the dependence of |*S*_11_|_*dB*_ only at *f*_1_ and *f*_2_. In contrast, (63) yields a more general solution set of *a* that provides information about all the maxima/minima between *f*_1_ and *f*_2_ as the even mode impedances (*Z*_1*e*_, *Z*_2*e*_, *Z*_3*e*_) vary.

#### Some Special Cases for Input RL

The following important points can be observed regarding the behavior of |*S*_11_|_*dB*_.

**Figure 4:**
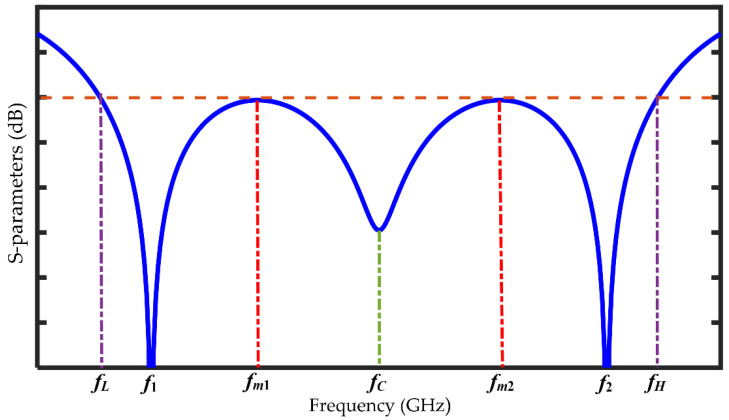
Specific frequency terms in S-Parameters.

**Figure 5:**
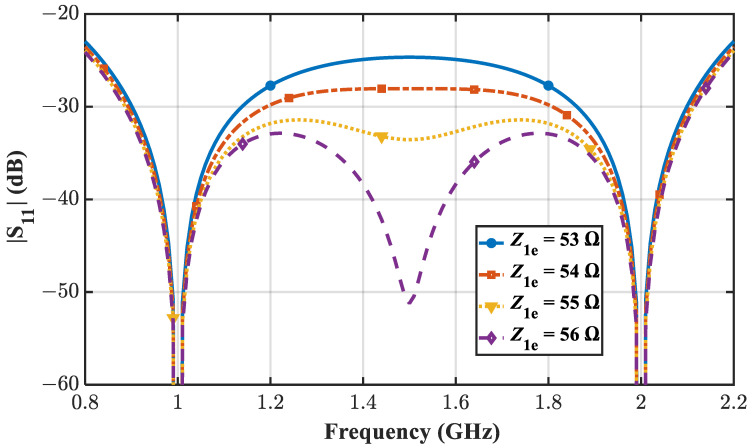
The behavior of |*S*_11_| between *f*_1_ and *f*_2_ for different *Z*_1*e*_ values.

**Figure 6:**
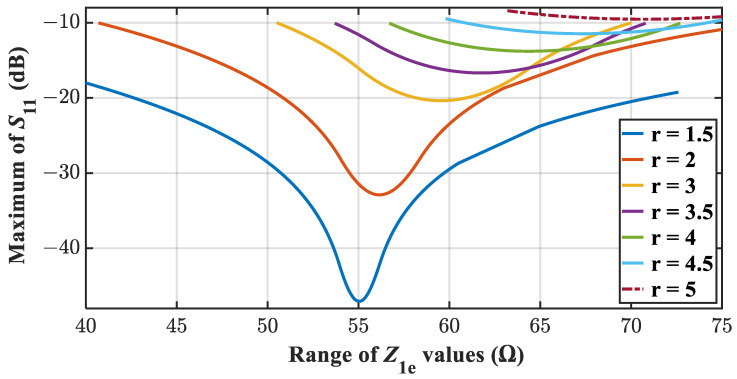
Variation of Maxima values of |*S*_11_(*f*_1_ < *f* < *f*_2_)| with *Z*_1*e.*_.

(i) Using (55) and (63), the common realizable solutions of *a*, irrespective of the value of *r*, can be found as:
  (69) $$a^{2}=\frac{k_{2}}{k_{3}}=\frac{1}{k_{1}}=\tan^{2}\theta$$
  These values of *a* correspond to the values of |*S*_11_|_*dB*_ at *f*_1_ and *f*_2_ where we will always get two minima, which is also evident from (6) owing to the dual-band principle. Now applying any suitable numerical analysis tool, we can calculate two other real values of *a* (*a_m_*_1_ and *a_m_*_2_). These values of *a* indicate two maxima of |*S*_11_|_*dB*_ at *f_m_*_1_ and *f_m_*_2_ where *f*_1_ < *f_m_*_1_ < *f_c_* and *f_c_* < *f_m_*_2_ < *f*_2_ and the center frequency, *f_c_* = 0.5(*f*_1_ + *f*_2_) = 0.5(*f_L_* + *f_H_*). These frequency levels are graphically defined in Figure 4.
  Furthermore, since *θ* ∝ *f*, the following equations can be derived with the help of (2) for which |*S*_11_|_*dB*_ becomes the maximum.
  (70) $$f_{m1}=\frac{f_{1}(1+r)}{\pi }\tan^{-1}a_{m1}$$
  (71) $$f_{m2}=f_{1}\left(1+r\right)-f_{m1}$$
  Some examples are shown in Table 1 where different solutions of *a* from (63) have been tabulated.
(ii) From (62), it can be further deduced that, for the minimum band-limited region (*f*_1_ < *f* < *f*_2_), we have the following cases.
  (72) $$\mathrm{Case}-1:\;\;\;\;\;\;\;\;\;\frac{k_{3}}{k_{6}}\geq \left|S_{11}\left(f\right)\right|\;\;\;\;\;\;\;\;\;\;$$
  For this case, we can obtain two minima at *f*_1_, *f*_2,_ and one maximum at *f_c_*. Thus, the achievable BW can be found at the |*S*_11_(*f_c_*)|_*dB*_ level which is also visible from Figure 5 (*Z*_1*e*_ = 53 Ω and 54 Ω as examples where |*S*_11_(*f_c_*)| = −25 dB and −28 dB, respectively).
  (73) $$\mathrm{Case}-2:\;\;\;\;\;\;\;\;\;\frac{k_{3}}{k_{6}}<\left|S_{11}\left(f\right)\right|\;\;\;\;\;\;\;\;\;\;$$
  For this case, we can obtain two minima at *f*_1_, *f*_2,_ and two equal maxima at *f*_m1_, *f_m_*_2_. Thus, the achievable BW can be found at the |*S*_11_(*f_m_*_1_)|_*dB*_ level which is also visible from Figure 5 (*Z*_1*e*_ = 55 Ω as an example where |*S*_11_(*f _m_*_1_)| = −31.5 dB).
  (74) $$\mathrm{Case}-3:\;\;\;\;\;\;\;\;k_{3}<<k_{6}\&k_{3}\approx 0\;\;\;\;\;\;\;\;\;\;$$
  For the above case, we will get the minimum possible value at *f_c_*, and this can be visualized from Figure 5 (*Z*_1*e*_ = 56 Ω as an example). Also in this case, we will get three minima at *f*_1_, *f_c_* and *f*_2_, and two equal maxima at *f_m_*_1_ and *f_m_*_2_. This is also the point beyond which the maxima of *S*_11_ cannot be lowered further by choosing any value of *Z*_1*e*_, *Z*_2*e*_, or *Z*_3*e*_. So, it will give the best possible BW with the lowest possible |*S*_11_|_*dB*_ and the achievable BW can be found at the |*S*_11_(*f_m_*_1_)|_*dB*_ level where |*S*_11_(*f _m_*_1_)| = −33 dB as seen for *Z*_1*e*_ = 56 Ω.
  Hence, from the value of *k*_3_/*k*_6_, we can decide which design parameters to choose to arrive at either of the above three cases. In a special case when *k*_3_ = 0, it can be inferred from (55) that
  (75) $$Z_{3e}Z_{1e}=\sqrt{2}Z_{0}Z_{2e}$$
  Consequently, choosing *Z*_2*e*_ = $\sqrt{2}$*Z*_0_ as a particular solution of (18) and using (75), we conclude the following.
  (76) $$Z_{2e}=\sqrt{Z_{3e}Z_{1e}}$$
  Thus, after finding *Z*_1*e*_ that ensures *S*_11_ = 0, Equation (76) can be used to find *Z*_3*e*_ which gives the best possible maxima of *S*_11_ between *f*_1_ and *f*_2_. This equation will not be applicable unless the best possible maxima of *S*_11_ is targeted.
(iii) Based on the variation of maxima of *S*_11_ versus *Z*_1*e*_ shown in Figure 6, it is apparent that the higher bandwidth level can be achieved from the lower values of *r*. In fact, for *r* = 5, it is not possible to achieve 10 dB return loss as depicted in Figure 6. Further, the best possible maxima can be achieved for a certain value of *Z*_1*e*_. From that value of *Z*_1*e*_ shown in Figure 6, we can calculate *Z*_2*e*_ and *Z*_3*e*_ from (18) and (12) respectively. It can be verified that these values of *Z*_1*e*_, *Z*_2*e*_, and *Z*_3*e*_ also satisfy (76).

### 3.4. Maximum Output Return Loss (RL) and Isolation between f_1_ and f_2_

A similar procedure is also followed to minimize |*S*_22_|_*dB*_ and |*S*_23_|_*dB*_ between *f*_1_ and *f*_2_. Since there are more free variables involved compared with |*S*_11_|_*dB*_ case, one can easily perform this analysis directly in MATLAB with the help of (40), (41), (44) and (54).

## 4. The Proposed Design Methodology

**Figure 7:**
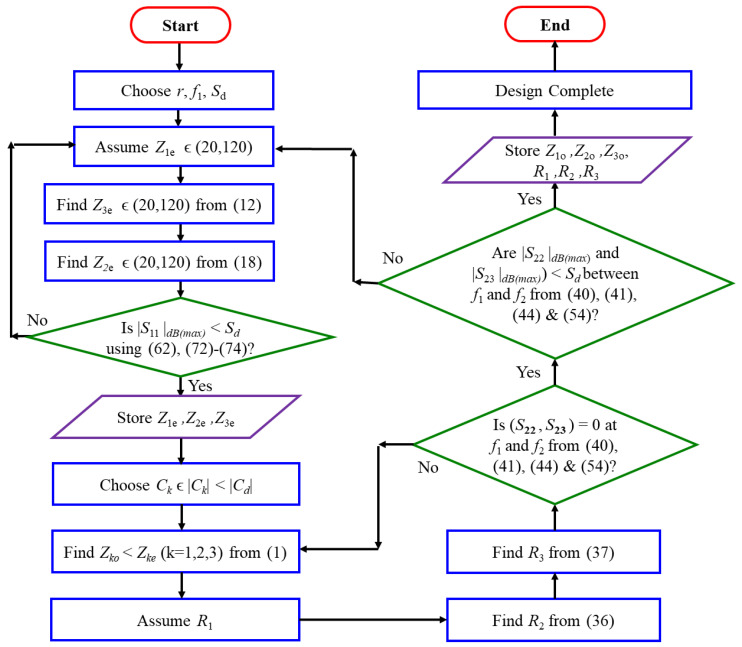
Flowchart of design steps.

To be physically realizable, coupled-line microstrip technology must have a certain specific value of coupling coefficient. We consider *C_d_* to be the limit of the coupling coefficient such that the actual coupling coefficient of each coupled-line section |*C_k_*| < |*C_d_*|. In addition, *S_d_* is the desired *S*-parameter value in dB to ensure the bandwidth requirement for wideband performance such that (|*S*_11_|_*dB*_ _(max)_, |*S*_22_|_*dB*_ _(max)_ and |*S*_23_|_*dB*__(max)_) < *S_d_* between *f*_1_ and *f*_2_. With these limits set, Figure 7 depicts the flowchart for the proposed analytical design methodology to find *R_k_*, *Z_ke_* and *Z_ko_* values (*k* = 1, 2, 3) that will ensure a wideband profile.

**Figure 8:**
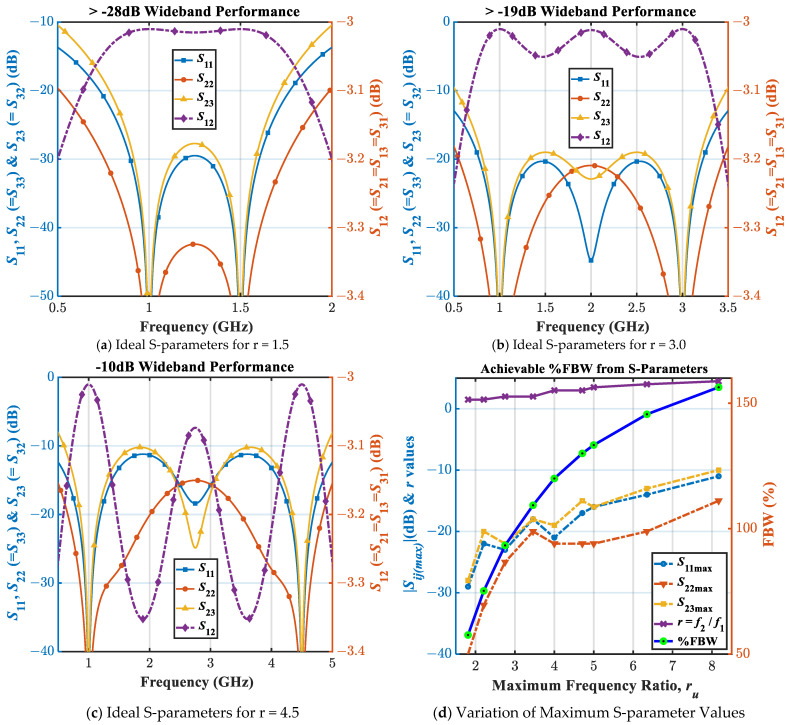
Ideal S-parameters with the variation of *r*, *r_u,_* and %FBW.

Following the above design methodology, some design examples for *r* = 1.5, 2, 3, 3.5, 4, and 4.5 are shown in Table 2. The ideal S-parameters corresponding to *r* = 1.5, 3, and 4.5 are plotted in Figure 8a–c.

We can observe the wideband performance of the proposed WPD from the graphical representations of the ideal S-parameters. From Figure 8a–c, it is inferred that with the increased value of *r*, the wideband performance deteriorates. Specifically, it is apparent that when *r* = 4.5, the return loss and isolation just touch the −10 dB level between *f*_1_ and *f*_2_ while resulting in a transmission ripple of less than 0.4 dB. Thus, it is theoretically possible to utilize the proposed design methodology to design the wideband WPD for *r* < 4.5.

Now, from the examples considered in Table 2 and the simulation results shown in Figure 8a–c, we can easily deduce the cases listed in Table 3 and the corresponding plot depicted in Figure 8d. They reveal the wideband operability for *r* < 4.5 and variation of % FBW, respectively, at different dB levels. We can also assess the associated % FBW for any set of calculated |*S*_11_|_*dB*_ _(max)_, |*S*_22_|_*dB*_ _(max)_ and |*S*_23_|_*dB*__(max)_. It must be emphasized that *r* = *f*_2_/*f*_1_ is the frequency ratio that is used only during the design process, since the design process utilizes the dual-band concept. Whereas, the actual bandwidth is determined by attainable band ratio *r_u_* = *f*_H_/*f*_L_, as depicted in Figure 1b. For instance, as illustrated in Table 3, for *r* = 3 we have the resonance frequencies at 1 GHz and 3 GHz with the attainable band ratio of 4.00 to 6.70 from 19 dB to 10 dB level (minimum of return loss and isolation) and 3.05 dB to 3.21 dB level (maximum insertion loss [1]), respectively. Since the operability in the wideband region is entirely based on the concept of the dual-band technique, it is also observed from Figure 8d that our proposed WPD can be designed theoretically with *r_u_* in the range of 1–8.2. Furthermore, it is observed that we can even theoretically achieve a maximum 157% fractional BW from this newly designed WPD.

It is apparent that for *r* > 4.5, the isolation becomes lesser than 10 dB between *f*_1_ and *f*_2,_ and therefore, the proposed WPD exhibits only the dual-band performance.

## 5. The Fabricated Prototype and Measurement Results

The proposed analytical design methodology is used to design a wideband WPD with *S_d_* < −20 dB and *C_d_* = −12 dB as a demonstration. If the resonance frequencies are chosen as *f*_1_ = 0.9 GHz and *f*_2_ = 1.8 GHz, then the calculated ideal design parameters using MATLAB are found to be as: *Z*_1*e*_ = 54.00 Ω, *Z*_2*e*_ = 68.04 Ω, *Z*_3*e*_ = 85.63 Ω, *Z*_1*o*_ = 35.00 Ω, *Z*_2*o*_ = 40.00 Ω, *Z*_3*o*_ = 45.00 Ω, *R*_1_ = 599.00 Ω, *R*_2_ = 37.87 Ω, and *R*_3_ = 8.05 Ω.

**Figure 9:**
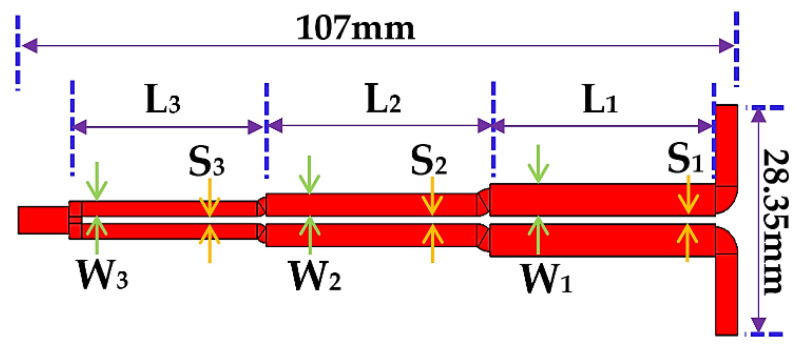
WPD EM modeling using ADS momentum.

**Figure 10:**
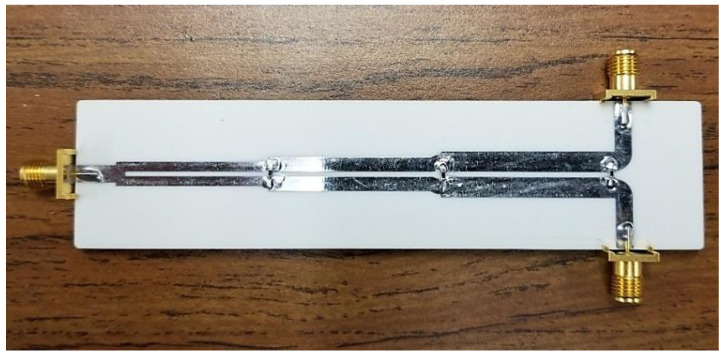
The fabricated prototype of the WPD.

Based on the analytical design, these values are subsequently used to implement and optimize the WPD in microstrip technology using the ADS design environment. The optimized (to tackle junction discontinuities) physical dimensions of the designed prototype are listed in Table 4. Based on these dimensions, the designed WPD electromagnetic (EM) model is shown in Figure 9 whereas the fabricated prototype is depicted in Figure 10. The Rogers’ RO4003C laminate is used with the following parameters: dielectric constant, ϵ_r_ = 3.55, substrate height = 1.524 mm, thickness = 35 µm and loss tangent, tanδ = 0.002. The final resistors for this prototype WPD are: 2*R*_1_ = 634 Ω, 2*R*_2_ = 95.3 Ω, and 2*R*_3_ = 24.9 Ω which correspond to commercially available values.

**Figure 11:**
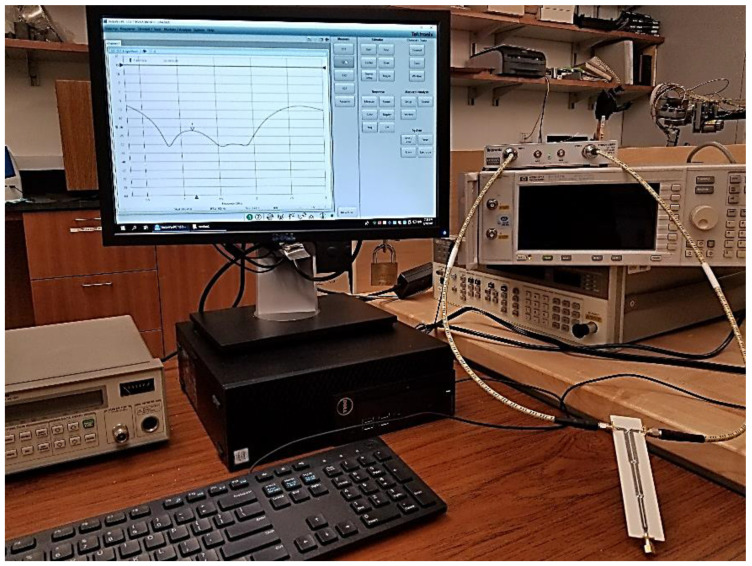
The measurement of the fabricated prototype using the Tektronix VNA.

**Figure 12:**
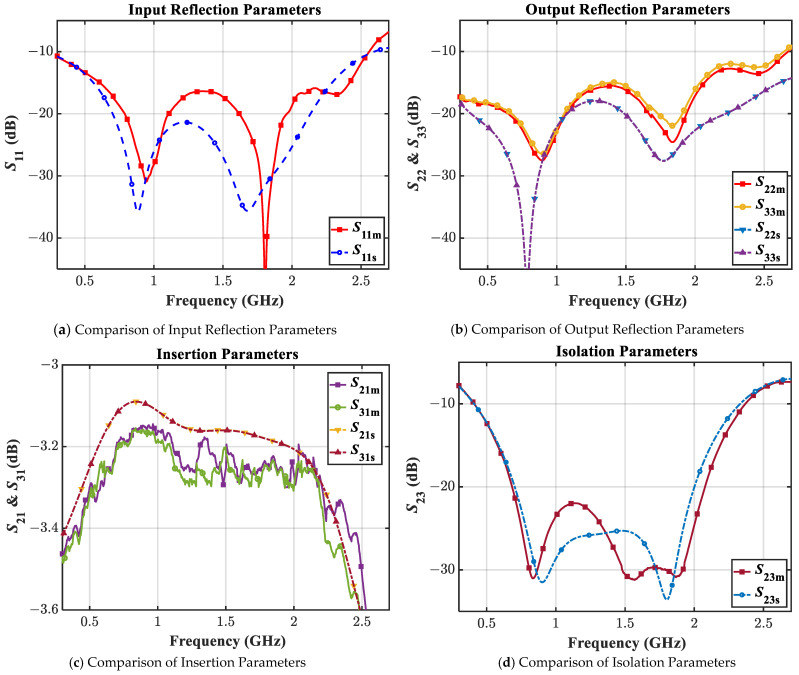
The EM simulated (subscript s) vs. the measured (subscript m) magnitudes of S-parameters.

**Figure 13:**
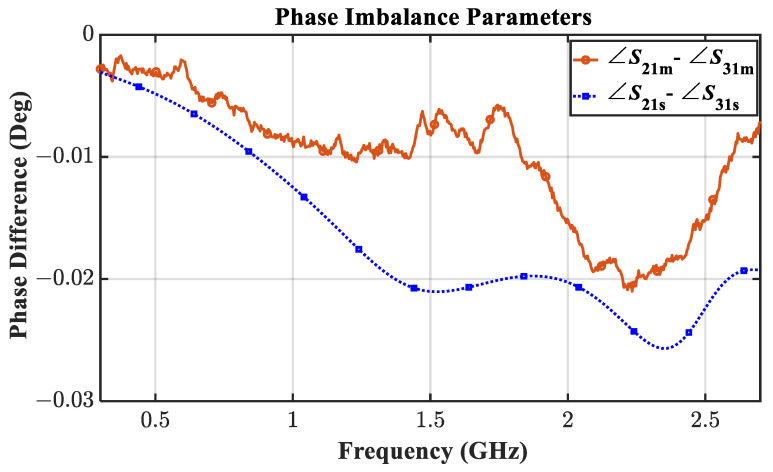
Simulated (subscript s) vs. measured (subscript m) phase imbalance between output ports.

The prototype is measured using the Tektronix TTR506A vector network analyzer (VNA) with a full two-port SOLT calibration as shown in Figure 11. The EM simulated and the measured results in the range of 0.3 GHz–2.7 GHz are compared in Figure 12 and Figure 13.

It is apparent from Figure 12a,b that the designed prototype has excellent reflection parameters maintaining an input return loss greater than 16.4 dB and an output return loss greater than 15 dB throughout the band from 0.595 GHz to 2.05 GHz. Besides, the transmission parameters are also found to demonstrate decent performance in Figure 12c ensuring an insertion loss of lesser than 3.30 dB, not far from the ideal 3 dB value in the aforesaid band. Moreover, as shown in Figure 12d and Figure 13, the measured isolation and phase imbalance of 22 dB (minimum) and value of 0.02 degrees (maximum), respectively, are remarkable. From the aforesaid band, the measured % FBW is 110% considering the −15 dB reference line whereas it becomes 141% considering a −10dB reference level from 0.41 GHz to 2.37 GHz, which is outstanding.

Though there is a slight mismatch observed between the EM simulated and the measured values, which normally occurs in any high-frequency design, possibly it is mostly due to the Panasonic isolation resistor’s non-idealities at high frequencies. Unfortunately, the model of these resistors is not available, so it was not possible to include their impact in the simulation. Nevertheless, the measured results show the outstanding performance of the WPD design which meets or exceeds all the goals that a practical WPD will require during deployment in the field.

Although the main advantage of the proposed design is its fully analytical and computer-friendly design methodology, to highlight the novelty of this work further, a comparison table is presented in Table 5 where some recent state-of-the-art works have been listed. It is conspicuous from the Table that the proposed WPD proves to be the best fit in terms of fulfilling the desired values of all the S-parameters in the wideband zone and maximizing the % FBW at the same time compared to all these previous works.

## 6. Conclusions

A completely new equations-based design approach for a three-section WPD has been discussed in this paper. The methodology started with deriving the required equations for the coupled transmission lines and then utilized the dual-band design technique to arrive at a robust design for a wide-band performance. The design procedure has been outlined through a compact flowchart which is followed to demonstrate some examples highlighting the capabilities of the proposed technique. Afterward, the fabricated prototype shows good agreement with the simulated data from the measurement results and substantiates the theoretical aspect. The biggest challenge of this design was to incorporate the junction discontinuities due to the use of coupled line with different widths, which needed an EM optimization. Nonetheless, the presented design equations will be the ideal choice for a computer-aided design of a miniaturized wideband WPD consisting of microstrip coupled-line.

## Figures and Tables

**Table 1:** Calculated real values of *a* from (63).

Band Ratio, *r*	Z*_ke_* Values, *k* ϵ {1, 2, 3}			Solution of *a*	Remarks on |*S*_11_|_*dB*_ from the Value of *a*
	*Z*_1*e*_ (Ω)	*Z*_2*e*_ (Ω)	*Z*_3*e*_ (Ω)		
2	56.1520	70.7107	89.0442	$\pm \sqrt{3}$	Minima at *f*_1_ & *f*_2_
				$\pm \sqrt{11}$	Maxima at *f_m_*_1_ & *f_m_*_2_
3	59.5380	70.7107	83.9807	±1	Minima at *f*_1_ & *f*_2_
				$\pm \sqrt{5}$	Maxima at *f_m_*_1_ & *f_m_*_2_
4	64.3670	70.7106	77.6800	$\pm \sqrt{5-2\sqrt{5}}$	Minima at *f*_1_ & *f*_2_
				$\pm 8\sqrt{7}/5\sqrt{5}$	Maxima at *f_m_*_1_ & *f_m_*_2_

**Table 2:** Circuit Parameters with the variation of *r*.

*r*	*Z*_1*e*_ (Ω)	*Z*_1*o*_ (Ω)	*Z*_2*e*_ (Ω)	*Z*_2*o*_ (Ω)	*Z*_3*e*_ (Ω)	*Z*_3*o*_ (Ω)	*R*_1_ (Ω)	*R*_2_ (Ω)	*R*_3_ (Ω)
1.5	60.00	37.00	81.26	42.00	99.04	50.00	929.00	31.26	5.01
2	58.30	34.80	73.38	40.00	92.45	48.00	500.00	34.52	5.00
3	59.00	36.00	70.72	37.00	83.22	41.00	910.00	84.20	23.45
3.5	60.00	36.00	71.45	38.00	78.64	44.00	1000.00	81.60	31.15
4	63.00	35.00	71.66	42.00	76.04	43.00	355.00	76.25	37.42
4.5	64.50	34.00	73.26	40.00	71.17	41.00	151.00	109.49	39.42

**Table 3:** Limitation on Wideband Performance.

Minimum Band Ratio, *R*	Minimum of Return loss & Isolation (dB)	Maximum Insertion loss (dB)	Operating Band Range, *f_L_* to *f_H_* (GHz)	Extra BW, *f_ex_* (GHz)	Attainable Band Ratio, *r_u_*
1.50	28	3.01	0.89–1.61	0.11	1.81
	10	3.20	0.48–2.02	0.52	4.21
2.00	20	3.02	0.80–2.20	0.20	2.75
	10	3.20	0.50–2.50	0.50	5.00
3.00	19	3.05	0.80–3.20	0.20	4.00
	10	3.21	0.52–3.48	0.48	6.69
3.50	16	3.11	0.75–3.75	0.25	6.35
	10	3.22	0.55–3.95	0.45	7.77
4.00	13	3.20	0.68–4.32	0.32	5.00
	10	3.22	0.57–4.43	0.43	7.18
4.50	10	3.35	0.60–4.90	0.40	8.17

**Table 4:** Physical Dimension of the Coupled T-lines.

	3rd Line (n = 3)	2nd Line (n = 2)	1st Line (n = 1)
W_n_ (in mm)	1.918	2.823	3.987
S_n_ (in mm)	0.854	0.919	0.999
L_n_ (in mm)	29.66	33.62	33.82

**Table 5:** Comparison of performance among this WPD and some other recent works.

Reference	Techniques/Topology	Band Ratio (*r_u_* = *f_H_*/*f_L_*)	Min. Input RL (dB)	Min. Output RL (dB)	Min. Isolation level (dB)	Max. Insertion level (dB)	Max. Phase Imbalance of Outport Ports (Degree)	FBW (%)
[24]	RLCT Isolation network	3.55	10	10	20	4.00	-	112.00
[25]	Frequency-selecting coupling structure	2.13	20	-	12	3.40	4.00	62.07
[26]	Parallel coupled filters	1.66	15	14	22	4.00	-	49.50
[27]	Single section optimized isolation network	2.20	19	20	19	3.20	-	75.00
[35]	Two open-circuited stubs and three coupled lines	1.90	10	10	10	-	2.50	62.00
[39]	Isolation stage shifting	2.08	-	-	17	3.90	-	70.20
[40]	Multilayer slot line structure	3.22	10	10	10	5.00	2.00	105.30
[41]	Hybrid Wilkinson and Gysel structure	1.78	16	17	20	4.40	1.70	56.00
[42]	LC ladder	3.08	18	17	18	3.90	-	102.00
[43]	Port matching using three coupled-line, one T-line and one isolation resistor	2.00	15	17	15	3.20	0.50	66.67
[44]	Quasi-coupled line with one open stub and two shorted stubs	3.19	15	15	15	3.66	6.80	104.50
[45]	Transversal filters with RLC network	2.57	15	15	17	-	1.60	87.80
[46]	Four T-lines, two stubs & five resistors	3.42	17.8	20	19.8	4.00	-	109.50
[47]	Port-to-Port isolation structure	2.28	10	10	17.5	3.70	1.50	78.00
[48]	Synthesis theory and optimization algorithm	1.92	21.1	19.2	18.5	3.10	1.20	63.00
This Work	Three Coupled T-Lines & three isolation resistors	3.45	16.4	15	22	3.30	0.017	110.11
		5.78	15.9	12	10	3.50	0.021	141.00

## Data Availability

Not applicable.
